# Commentary: COVID-19 Mortality: A Matter of Vulnerability Among Nations Facing Limited Margins of Adaptation

**DOI:** 10.3389/fpubh.2021.642825

**Published:** 2021-04-16

**Authors:** Roxane Borgès Da Silva, Georges Borgès Da Silva

**Affiliations:** ^1^École de santé publique, Université de Montréal, Montréal, QC, Canada; ^2^Centre de Recherche en Santé Publique (CReSP), Montréal, QC, Canada; ^3^Center for Interuniversity Research and Analysis on Organizations, Montreal, QC, Canada; ^4^Bibliothèque de Santé, La Seyne sur mer, France

**Keywords:** COVID-19, GDP per capita, mortality rate, demography, public health

## Introduction

De Larochelambert et al. (1) claimed to find a correlation between GDP/Capita and COVID-19 mortality, and that they also found such correlation of mortality with many other variables (partly in correlation with GDP/Capita). Economic development and associated growth in GDP tend to increase comorbidities, they argued, and therefore the most severe forms of COVID-19.

Researchers from developed countries should think less schematically of Africa (and other developing countries) and be more open-minded and scientifically careful. On the one hand, developing countries also have significant comorbidity rates, on the other hand, their health statistics may be unreliable.

Indeed, Africans, in particular, are not free of comorbidities. Research has shown a hypertension prevalence rate higher than that of the population of European origin (2). Similarly, obesity affects up to 30% of the urban adult population. According to the WHO, across 36 African countries, 23.8% of the women are overweight, with the rate exceeding 40% in Gabon, Ghana and Lesotho, and reaching a maximum of 50.6% in Swaziland (3). The WHO estimates the prevalence of diabetes at ~5% of the African population (4).

The low COVID-19 mortality rate in emerging countries is usually attributed to other possible factors than that of the prevalence of comorbidities: endemic diseases reduce life expectancy and so the average age of the population is very low (5). As a result, a large part of the population never reaches the peak age of susceptibility to COVID-19. Furthermore, lower population density and the fact that few seniors live in care homes may also be factors in the low mortality rate (6). At the moment, unpublished studies are focusing on the possibility of cross-protective immunity with other coronaviruses common in Africa (HCoV-OC43, HCoV-HKU-1, HCoV-NL63, HCoV-229).

## Is There A Relationship Between A Country's Gross Domestic Product (GDP) and its COVID-19 Mortality Rate?

**Figure 1:**
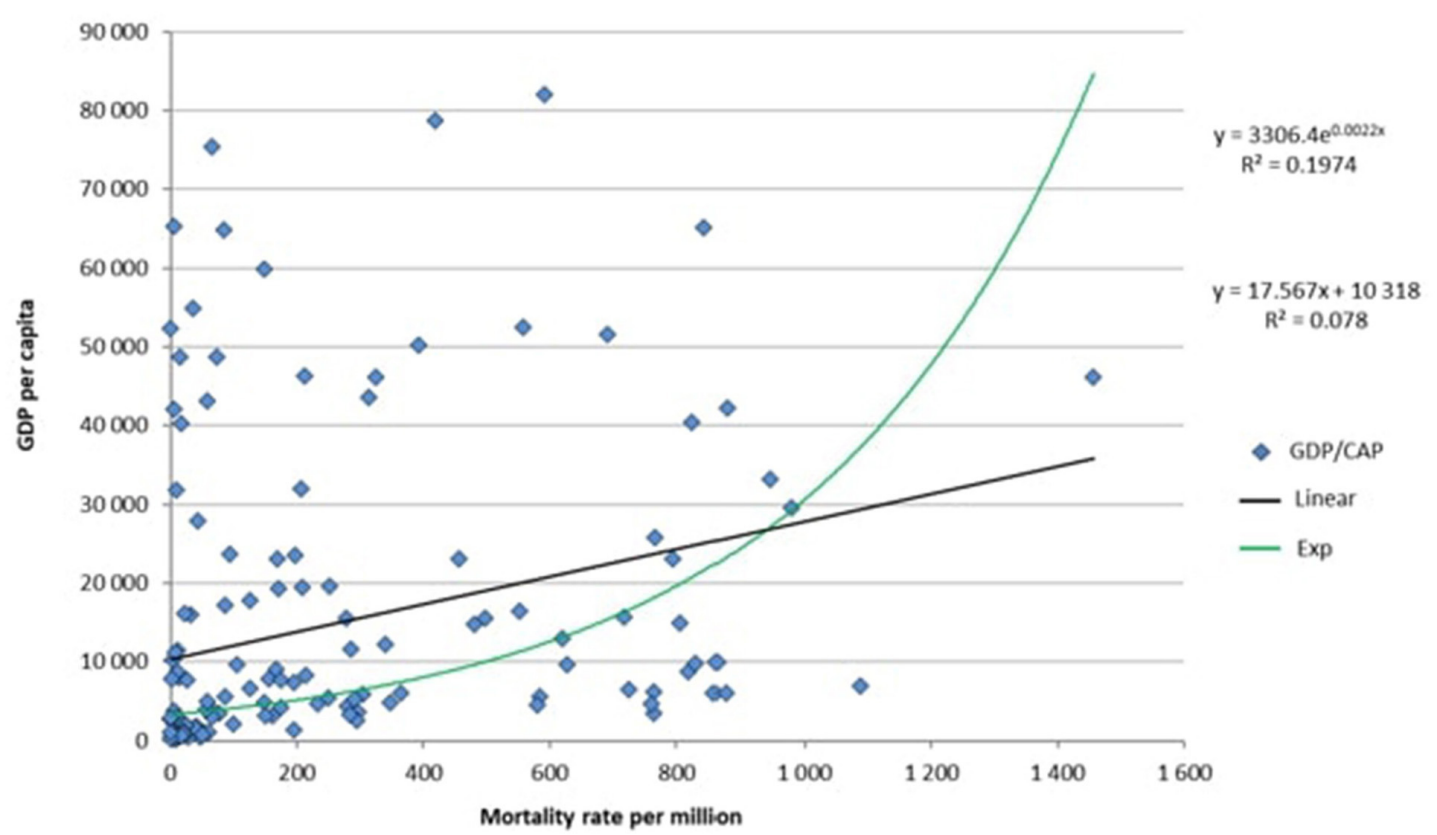
Relationship between GDP per capita and mortality rate per one million inhabitants, for 150 countries with populations of over one million.

If comorbidities are not at the root of the difference in COVID-19 mortality rates, is there really a relationship between GDP per capita and the COVID-19 mortality rate? Like De Larochelambert et al., we have found a significant statistical relationship between these two variables. Our Figure 1 shows the theoretical straight line and curve that could represent this relationship (In our analysis we found that *p* = 0.0005.) The figure concerns 150 countries having populations of over one million (data for countries with smaller populations are subject to statistical fluctuation). For each country, we crossed GDP per capita from 2019 (7) with its COVID-19 mortality rate for one million inhabitants as of December 11, 2020 (8).

But this statistical result is strongly impacted by a bias stemming from a confounding variable: the reliability rate of each country's public health data records. In all likelihood, there is a strong statistical relationship between the degree of reliability of the data and the GDP per capita (the lower a country's GDP per capita, the less able it will be to produce reliable public health statistics). That explains the major clustering of data points close to the origin (zero) of the coordinate axes. The cluster is made up of very-low-GDP countries that are not able to ensure the accuracy of their public health data, nor provide their inhabitants with easy access to PCR testing, especially in cases where the country is in turmoil. This clustering near zero is a major factor in statistical significance and deprives the statistical analysis of all credibility. It is also important to consider that each country has its own method of counting deaths by COVID-19, which includes one or more or all possible places where deaths occur (in a hospital, seniors' residence, at home).

To make their data more reliable, the authors excluded countries that reported fewer than 10 deaths by covid-19. But this restriction seems insufficient. In our calculation, we included only countries having a GDP per capita of greater than US$1200. The last country to make this cut-off was Benin, which had a COVID-19 mortality rate of four for every one million inhabitants. On this basis, the following countries were excluded: Lesotho, Tanzania, South Sudan, Nepal, Equatorial Guinea, Guinea, Yemen, Mali, Tajikistan, Ethiopia, Rwanda, Uganda, Burkina Faso, Haiti, Gambia, Chad, Guinea-Bissau, Togo, Liberia, Niger, Democratic Republic of Congo, Madagascar, Sierra Leone, Afghanistan, Mozambique, Central African Republic, Sudan, Malawi, Burundi, Somalia.

**Figure 2:**
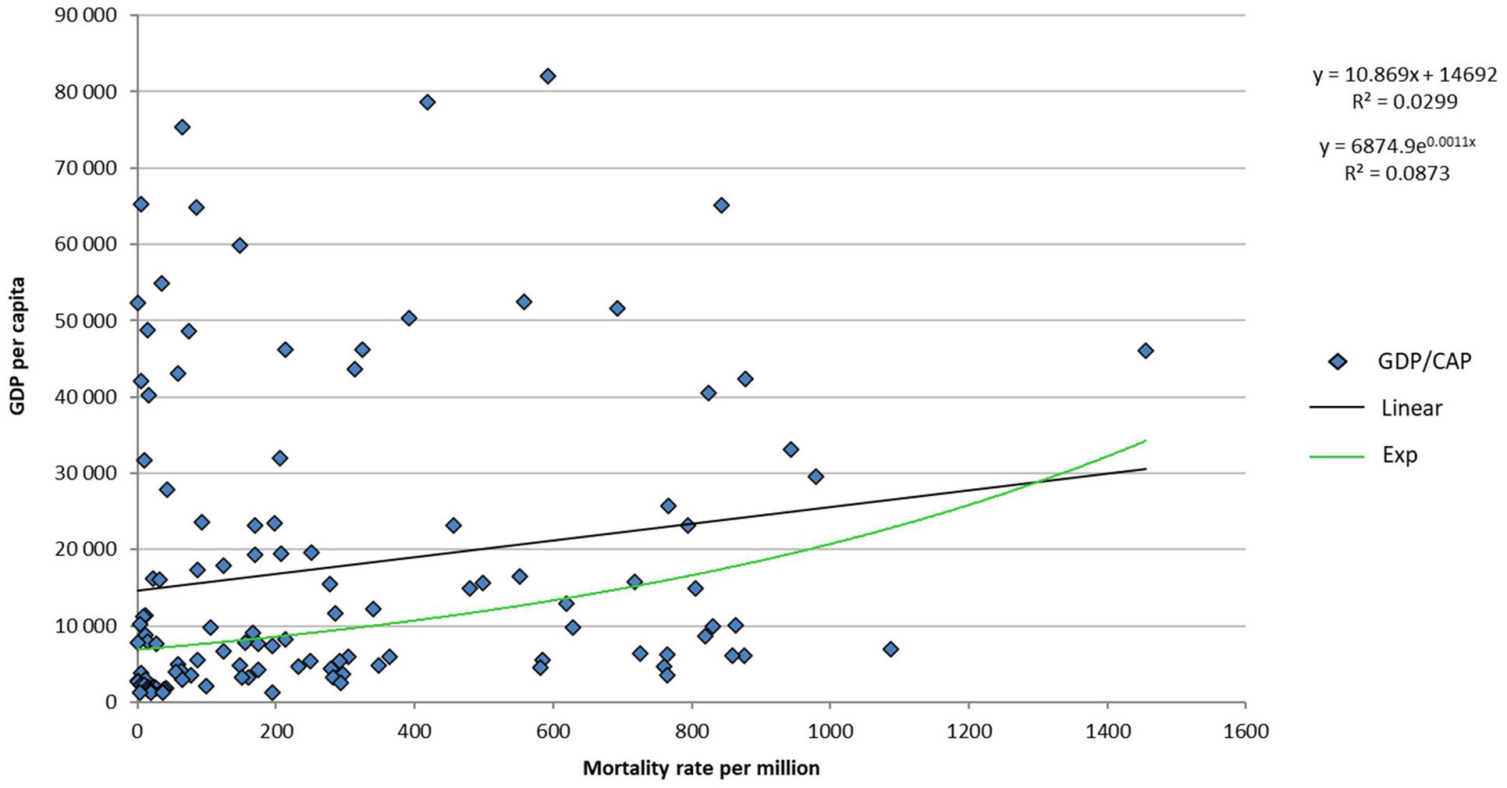
Relationship between GDP per capita and mortality rate per million inhabitants for 120 countries with more than one million inhabitant and a GDP per capita greater than $1,200.

When restricted to these 120 countries, the statistical analysis did not reveal any relationship between GDP per capita and COVID-19 mortality rate. With a base GDP-per capita level of US$1200 dollars, a significant statistical relationship (at 5%level) between GDP per capita and COVID-19 mortality rate can no longer be found (*p* = 0.0588) (Figure 2). Note that for the 107 countries with a GDP per capita greater than $2,000, the alpha statistical risk is amplified (*p* = 0.29).

## Discussion

While the choice we made, certainly involves a loss of information, the loss is offset by the greater reliability of the retained data.

The statistical relationship identified by the authors omits consideration of a major confounding variable that is strongly linked to GDP when the latter is low: the reliability rate of each country's public health data records. The conclusion regarding the relationship between GDP and the COVID-19 mortality rate is biased and unclear with respect to its application.

## Author Contributions

All authors listed have made a substantial, direct and intellectual contribution to the work, and approved it for publication.

## Conflict of Interest

The authors declare that the research was conducted in the absence of any commercial or financial relationships that could be construed as a potential conflict of interest.
